# Transperitoneal Robot-Assisted Radical Prostatectomy Should Be Considered in Prostate Cancer Patients with Pelvic Kidneys

**DOI:** 10.1089/cren.2016.0006

**Published:** 2016-02-01

**Authors:** Sophie Plagakis, Darren Foreman, Peter Sutherland, Andrew Fuller

**Affiliations:** ^1^ Department of Urology, Repatriation General Hospital, Daw Park, Australia.; ^2^ Department of Urology, Royal Adelaide Hospital, Adelaide, Australia.

## Abstract

We highlight two cases of transperitoneal robot-assisted radical prostatectomy (RARP) in patients with pelvic kidneys because of congenital development and renal transplant. These uncommon cases present a challenge to the surgeon contemplating surgery because of access and anomalous vascular and ureteral anatomy. We describe the technical considerations that are paramount in effectively completing transperitoneal RARP, and believe it should be considered as a treatment option in men with pelvic kidneys.

## Introduction and Background

Congenital pelvic kidneys are an uncommon incidental finding, with a reported incidence of ∼1 in 3000. Renal transplants are also uncommon, with less than 1000 renal transplants performed in Australia in 2014.The vast majority have a pelvic location. Both situations present a challenge to the surgeon contemplating a radical prostatectomy for prostate cancer, because of surgical access and anomalous vascular and ureteral anatomy.

We describe two cases of patients with pelvic kidneys who underwent robot-assisted radical prostatectomy (RARP) for localized prostate cancer through a six-port transperitoneal approach.

## Case Report 1

**FIG. 1.**
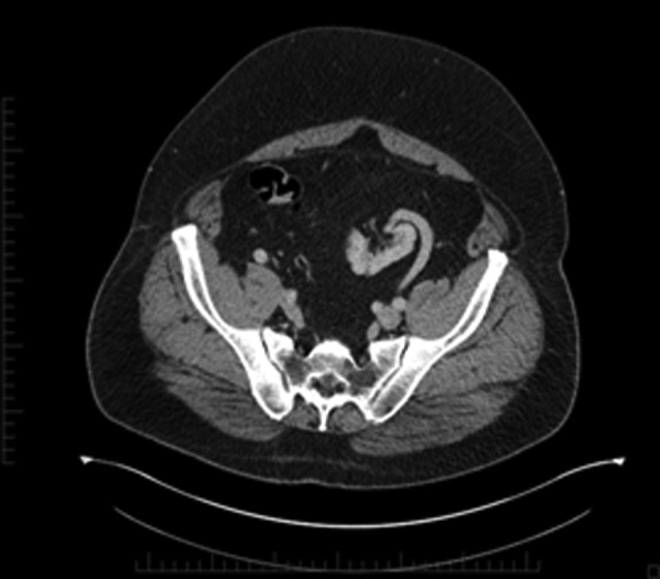
Case 1—CT urogram; axial image showing the medial location of a congenital pelvic kidney, which is rotated with renal vein in the lateral position.

**FIG. 2.**
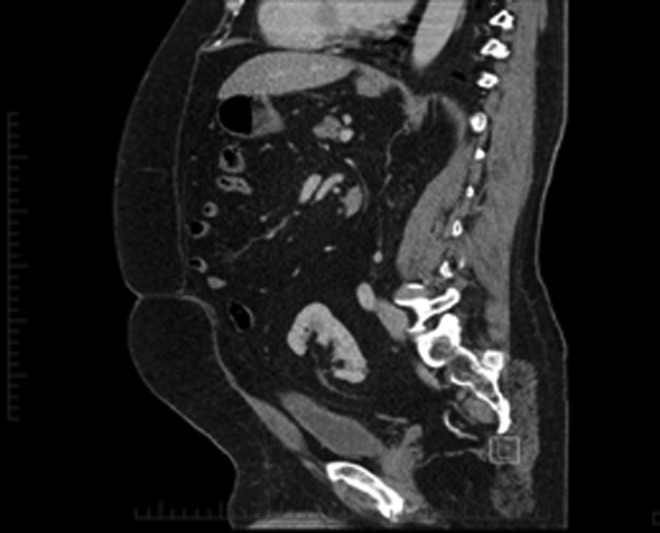
Case 1—CT urogram; sagittal image showing the proximity of ureter to the posterior aspect of the bladder in a congenital pelvic kidney.

A healthy 62-year-old man was referred for prostate biopsy because of a prostate-specific antigen (PSA) of 6.1 ng/mL. He was found to have a well-differentiated carcinoid tumor of the duodenum in 2012 that was metastatic to local lymph nodes and liver segment VI. His disease was stable, without the need for further surgery or oral medication, and he was monitored by a Medical Oncologist. He had no family history of prostate cancer, and digital rectal examination revealed a generally firm gland with no palpable nodules. Transrectal prostate biopsy confirmed Gleason 3 + 3 = 6 adenocarcinoma in three of seven cores from the right lobe. Whole body bone scan (WBBS) showed no metabolically active bone lesions. CT scan (Figs. 1 and 2) and MRI of the abdomen and pelvis showed a congenital pelvic kidney in his left pelvis with no lymphadenopathy or evidence of extraprostatic disease. RARP was performed on a Da Vinci S machine (Intuitive Surgical) by utilizing a bilateral nerve sparing technique without pelvic lymph node dissection. The console time was 95 minutes with an estimated blood loss of 150 mL. He was discharged on postoperative day 1 and there were no perioperative complications. Histopathology report confirmed Gleason 3 + 3 = 6 disease in the right lobe with no extraprostatic extension or seminal vesicle invasion (pT2c), clear surgical margins, and an estimated tumor volume of 4.7 cc. PSA became undetectable and the patient needed no further therapy. At 6 weeks postsurgery, his 24-hour pad weight showed urine loss of 50 mL. He no longer needed the use of continence pads at 3 months postsurgery. He regained erectile function satisfactory for intercourse with the use of sildenafil on demand by 6 months after surgery. He has been followed up for 12 months since his operation.

## Case Report 2

A 60-year-old renal transplant recipient was referred for consideration of RARP by a Urologist who was not comfortable offering open prostatectomy. He had already been considered for low-dose rate seed brachytherapy by a Radiation Oncologist. He developed end-stage renal failure secondary to IgA nephropathy in 1989, and underwent a renal transplant in 1990, which failed in 1995. A second renal transplant was performed in 1996, which was situated in the left pelvis and was functioning well at the time of prostate cancer diagnosis (creatinine 104, eGFR >60 mL/minute). He remained on immunosuppressive therapy. There was no family history of prostate cancer.

**FIG. 3.**
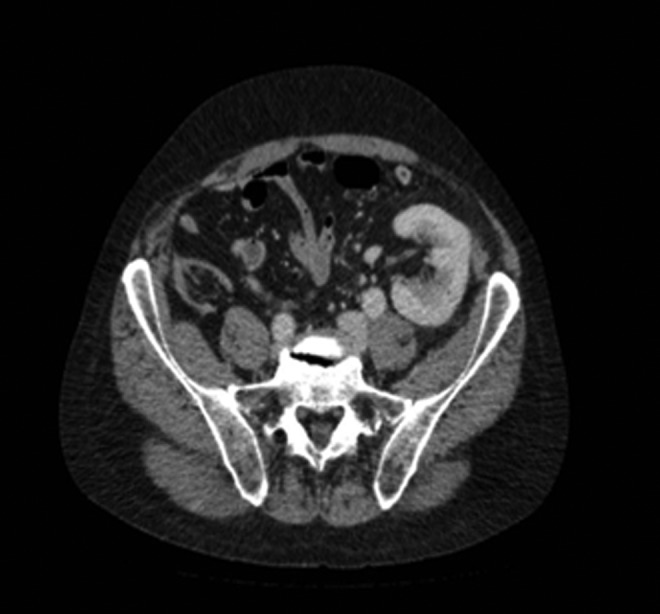
Case 2—CT urogram; axial image with renal transplant located lateral in pelvis.

**FIG. 4.**
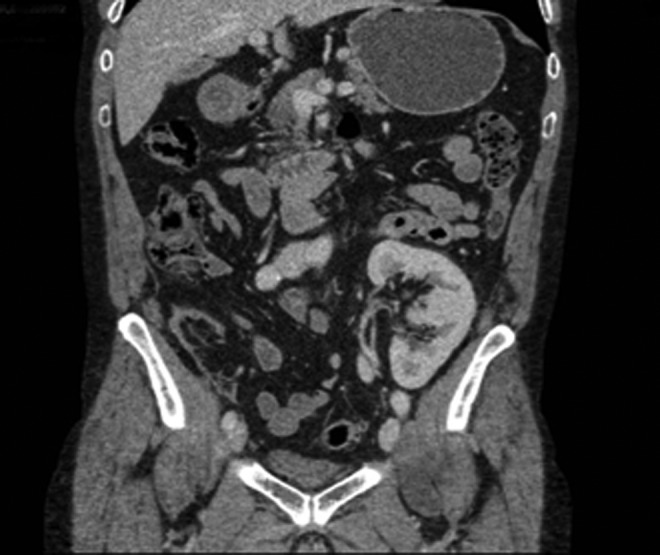
Case 2—CT urogram; coronal image showing the lateral pelvic position of the renal transplant.

His PSA was 13 ng/mL. Digital rectal examination was unremarkable and transrectal prostate biopsies confirmed widespread Gleason 3 + 3 = 6 disease. Staging WBBS, CT (Figs. 3 and 4), and MRI excluded any metabolically active bone lesion, lymphadenopathy, or extraprostatic extension. After review of radiology imaging and discussion with the Renal Transplant Surgeon, a bilateral nerve sparing RARP was performed on a Da Vinci standard machine (Intuitive Surgical) after small bowel adhesiolysis, without a pelvic lymph node dissection. Console time was 139 minutes with an estimated blood loss of 190 mL. He had an uneventful postoperative course and was discharged on day 2, after an additional day of monitoring by the Renal Physicians. His urine output remained more than 30 mL/hour after surgery and his creatinine was elevated at 138 (eGFR 49 mL/minute) at discharge. Histopathology report showed Gleason 3 + 4 = 7 disease in both lobes with no extraprostatic extension or seminal vesicle invasion (pT2c), clear surgical margins, and an estimated tumor volume of 6.6 cc. PSA became undetectable after surgery and the patient needed no further therapy. His renal function returned to baseline within 2 weeks of surgery. It has been 10 years since surgery and he is continent and has poor erectile function that responds to intracavernosal injection therapy, which was also used before his operation.

## Literature Review

Prostate cancer is more common in renal transplant recipients than in the general population, although the incidence remains low.^1^ Transplant recipients are chronically immunosuppressed and have well-established oncology surveillance protocols that aid early diagnosis of localized disease. Surgery remains the preferred treatment option for these patients and open radical prostatectomy has been established as a safe and effective treatment in the literature.^1,2^ RARP has been described as a safe procedure in patients with kidney transplants in several series.^1–3^ Key points raised by Jhaveri et al. included taking every precaution to identify and protect the ureter and vessels of the transplanted kidney during the pelvic lymph node dissection. The authors advocated the use of an extended length bariatric port on the ipsilateral side of the pelvic kidney to reduce instrument activity over the graft. It was also established that the standard laparoscopic pneumoperitoneum pressures of 15–18 mmHg are safe and did not induce hypoperfusion in the graft.

Extraperitoneal RARP has been described in a case of congenital pelvic kidney.^4^ The extraperitoneal approach was completed effectively without deviation from the standard surgical technique, and the patient had an unremarkable postoperative course.

## Comment

Standard robotic port placement for transperitoneal RARP was used for both patients. Open cutdown at the midline supraumbically and placement of a Hasson port allowed for laparoscopy and insertion of further ports under direct vision. Pneumoperitoneum pressure was set at 12 mmHg. No additional equipment was used in our series to improve observation of the ureter; however, placement of a ureteral catheter has been described in the literature to assist with identification. The reimplanted ureter of the transplant patient was not encountered during bladder mobilization, and the ureter inserted normally at the trigone in the pelvic kidney patient, necessitating no change to the standard dissection technique. Bladder neck anastomosis was performed using the van Velthoven technique with a 2/0 monofilament suture.

In our experience, the following technical considerations are paramount for effective completion of RARP in patients with a pelvic kidney.

1. *Careful preoperative review of imaging* to identify the anomalous ureteral and vascular anatomy.
2. *Gain initial access in the midline using an open Hasson technique* to identify adherent structures and location of the pelvic kidney.
3. *Perform a systematic pelvic laparoscopy* at the beginning of the procedure to correlate clinical findings with imaging.
4. *Place ports under direct vision with alteration of port position* depending on location of the pelvic kidney. Ports may need to be placed slightly cephalad or lateral on the ipsilateral side to provide additional working space around the pelvic kidney and to avoid instruments that may injure the pelvic kidney inadvertently.
5. *Consultation with the transplant surgeon* for renal transplant recipients.

## Conclusion

By adopting the technical considerations outlined, transperitoneal RARP is technically feasible and associated with satisfactory oncologic outcomes in our small series. It should be considered as a treatment option in men with pelvic kidneys.

## Abbreviations Used

CT: computed tomography
MRI: magnetic resonance imaging
PSA: prostate-specific antigen
RARP: robot-assisted radical prostatectomy
WBBS: whole body bone scan
